# Characterization of the enhancement of zero valent iron on microbial azo reduction

**DOI:** 10.1186/s12866-015-0419-3

**Published:** 2015-04-10

**Authors:** Yun Fang, Meiying Xu, Wei-Min Wu, Xingjuan Chen, Guoping Sun, Jun Guo, Xueduan Liu

**Affiliations:** School of Minerals Processing and Bioengineering, Central South University, 410083 Changsha, China; Guangdong Provincial Key Laboratory of Microbial Culture Collection and Application, Guangdong Institute of Microbiology, 510070 Guangzhou, China; State Key Laboratory of Applied Microbiology Southern China, 510070 Guangzhou, China; Key Laboratory of Biometallurgy of Ministry of Education, 410083 Changsha, China; Department of Civil and Environmental Engineering, Center for Sustainable Development and Global Competitiveness, Codiga Resource Recovery Center, Stanford University, Stanford, CA 94305 USA

**Keywords:** Azo reduction, *Shewanella decolorationis* S12, Zero valent iron (Fe^0^)

## Abstract

**Background:**

The microbial method for the treatment of azo dye is promising, but the reduction of azo dye is the rate-limiting step. Zero valent iron (Fe^0^) can enhance microbial azo reduction, but the interactions between microbes and Fe^0^ and the potential mechanisms of enhancement remain unclear. Here, *Shewanella decolorationis* S12, a typical azo-reducing bacterium, was used to characterize the enhancement of Fe^0^ on microbial decolorization.

**Results:**

The results indicated that anaerobic iron corrosion was a key inorganic chemical process for the enhancement of Fe^0^ on microbial azo reduction, in which OH^−^, H_2_, and Fe^2+^ were produced. Once Fe^0^ was added to the microbial azo reduction system, the proper pH for microbial azo reduction was maintained by OH^−^, and H_2_ served as the favored electron donor for azo respiration. Subsequently, the bacterial biomass yield and viability significantly increased. Following the corrosion of Fe^0^, nanometer-scale Fe precipitates were adsorbed onto cell surfaces and even accumulated inside cells as observed by transmission electron microscope energy dispersive spectroscopy (TEM-EDS).

**Conclusions:**

A conceptual model for Fe^0^-assisted azo dye reduction by strain S12 was established to explain the interactions between microbes and Fe^0^ and the potential mechanisms of enhancement. This model indicates that the enhancement of microbial azo reduction in the presence of Fe^0^ is mainly due to the stimulation of microbial growth and activity by supplementation with elemental iron and H_2_ as an additional electron donor. This study has expanded our knowledge of the enhancement of microbial azo reduction by Fe^0^ and laid a foundation for the development of Fe^0^-microbial integrated azo dye wastewater treatment technology.

**Electronic supplementary material:**

The online version of this article (doi:10.1186/s12866-015-0419-3) contains supplementary material, which is available to authorized users.

## Background

Azo dyes are widely used in the textiles, leather, plastics, cosmetics, and food industries, with a global annual production of more than 5,000 tons. Approximately 10% of azo products are discharged into the environment, resulting in a negative impact on the environment and human health because most azo dyes are carcinogenic, teratogenic, and highly persistent in the environment [1-3]. Conventional physicochemical methods for treating azo dyes have severe limitations, including incomplete removal, formation of hazardous products, and high operation costs, and biological techniques enable complete mineralization of the azo dye in a more environmentally friendly and cost-effective manner [4,5]. Azo dyes are not readily degraded by biological methods under aerobic conditions, and thus, they are normally decolorized by reductive cleavage of the azo bonds (−N = N-) under anaerobic conditions and then converted to aromatic amines, which are subsequently mineralized aerobically [6,7]. The decolorization process is typically rate-limiting, which hinders the biological treatment of azo dyes [8].

Zero valent iron (Fe^0^) can enhance anaerobic microbial azo reduction, but the exact mechanism of Fe^0^-assisted microbial reduction remains unclear [9-11]. Because azo dyes are decolorized by functional microorganisms, characterizing the Fe^0^-assisted decolorization of azo dyes using a pure decolorizing bacterium may provide some exact information about the reaction mechanism, and understanding how the microbes interacts with Fe^0^ will facilitate the elucidation of the mechanisms of enhancement and optimize the biodecolorization process.

Fe^0^ is a mild reducer and can chemically reduce several azo dyes under acidic and neutral conditions [12,13]. Furthermore, iron is an essential element for microbial survival because the active sites of diverse vital enzymes and proteins contain iron [14,15]. Microorganisms may regulate the iron redox reaction by cellular assimilation of iron. Meanwhile, Fe^0^ can produce cathodic H_2_ during the anaerobic corrosion process, and H_2_ is a favorable electron donor for microbial azo reduction [14,16-18]. According to the following equation

1 $$ {\mathrm{Fe}}^0 + 2{\mathrm{H}}_2\mathrm{O}\ \to\ {{\mathrm{Fe}}^2}^{+}+{\mathrm{H}}_2+2{\mathrm{OH}}^{\hbox{-} } $$

Fe^2+^, OH^−^, and H_2_ are the products of the anaerobic corrosion process [19]. The electrode potential of the redox couple Fe^2+^/Fe^0^ is −0.44 V, and hydrogen production from corrosion exhibits autocatalytic behavior, attaining a maximum rate of 1.9 mol kg^−1^ d^−1^ over 2 d of reaction in a study by Reardon *et al.* [20]*.* Based on these knowledge, it is hypothesized that anaerobic Fe^0^ corrosion may be accelerated in the azo dye biodecolorizing system, then (i) the microenvironmental conditions are altered to produce more favorable redox/pH conditions for the growth of microbes; (ii) the additional electron donor (H_2_) from Fe^0^ corrosion facilitates a greater microbial biomass yield; and (iii) the activity of the azo-reducing bacteria is stimulated by the supplementary elemental iron, resulting in the acceleration of the azo bioreduction.

To test these hypotheses, *S. decolorationis* S12, an azo-reducing bacterium isolated from a textile wastewater treatment system [21,22], was used as a model organism to characterize the enhancement of microbial azo reduction by Fe^0^ in this study. Specifically, we investigated (i) the effect of Fe^0^ dosage on the decolorization rate, pH change, and H_2_ release to determine whether Fe^0^ affects azo reduction by strain S12 indirectly; (ii) the morphology of Fe solids outside and inside the cells to determine whether a direct interaction between microbes and Fe^0^ occurs in the decolorization process; and (iii) the effect of Fe^0^ addition on the ratio of live versus dead cells and protein contents to determine whether the addition of Fe^0^ influences the growth and survival ability of strain S12. This study provides new insights into our understanding of the interactions between Fe^0^ and microbial cells in the decolorization process and lays a foundation for further optimization of Fe^0^-microbial integrated processes for efficient azo dye treatment.

## Methods

### Chemicals, organism, media, and cultivation

Amaranth (Am), a typical water-soluble azo dye, was purchased from Sigma (St. Louis, MO, USA). *S. decolorationis* S12 is a rod-shaped, gram-positive facultative bacterium that was isolated from the activated sludge of a textile-printing wastewater treatment plant by Xu *et al*. [22].

Strain S12 was cultivated by transferring a single colony to a 100-mL conical flask containing 50 mL of Luria-Bertani medium (10 g/L peptone, 5 g/L yeast extract, 10 g/L NaCl), which was then incubated in a shaking incubator (160 rpm) at 30°C. The cells were harvested in the middle of exponential growth phase (approximately 8 h) by centrifugation, washed twice with phosphate buffer (0.1 M, pH 7.4), and re-suspended in the buffer prior to inoculation. The cells were inoculated into a defined medium (pH 7.0) containing Na_2_HPO_4_, 7.64 g/L; KH_2_PO_4_, 3.00 g/L; NH_4_Cl, 0.50 g/L; NaCl, 1.00 g/L; sodium lactate, 5.00 mM; yeast extract, 0.5 g/L; and amaranth, 1.0 mM. The initial cell density was approximately 10^7^ CFU/ml (the protein mass was approximately 0.023 mg/mL). The cells were statically cultivated at 30°C in an anaerobic workstation (BugBox, Ruskinn Technologies). Standard anaerobic technique was used throughout the study for anaerobic cultivation as previously described [23]. The medium was prepared by adding concentrated stock solutions containing various medium components in O_2_-free distilled water. The medium was then bubbled with N_2_ gas for 10 min to remove residual air from the head space. All gases used were passed through a 0.2-μm filter prior to use. All batch experiments were conducted in 100-mL serum bottles with a culture medium volume of 40 mL under anaerobic conditions. Three independent experiments with triplicate bacterial samples were performed in each experiment.

Micrometer-sized iron (Fe^0^) particles (mean size of 18.51 μm) were obtained from Tianjin Guangfu Technology and Development Co., Ltd., China, and were pretreated by rinsing with 1 M HCl for 3 min, followed by washing with distilled water for 1 min. Different dosages of Fe^0^ (0, 10, 20, 30, 40, 60 mM) were added to the defined medium (40 mL) to examine the effect of Fe^0^ dosage on the decolorization reaction. The cell-free abiotic control (no strain S12 cells) received 60 mM Fe^0^. For the other tests, the dosage of Fe^0^ particles was maintained at 60 mM. Before and after decolorization (a reaction period of approximately 30 h), the size (hydrodynamic diameter) of the particles was determined using dynamic light scattering in a laser particle size analyzer (Eyetech, Ankersmid, USA).

### Growth and activity assays of microbial cells

Cells were grown in medium with and without 60 mM Fe^0^ for 12 h and then collected for cell yield and activity assays, including protein content and live/dead ratio. The protein content was determined by the Coomassie brilliant blue method [24] with slight modification. Briefly, NaOH solution (1 M, 40 mL) was added to the serum bottle containing the cell culture in 40 mL of medium, and the mixture was incubated in a water bath for 10 min at 95-99°C with gentle end-over-end inversion every 2 min. After centrifugation at 12,000 × *g* for 5 min, the supernatant was collected in a new centrifuge tube at room temperature and reacted with Bradford solution containing 0.01% (w/v) Coomassie brilliant blue G-250, 4.7% (w/v) ethanol, and 8.5% (w/v) phosphoric acid for 10 min prior to measuring the absorbance at 595 nm. Bovine serum albumin was used as a protein standard.

A LSM700 laser scanning confocal microscope (Zeiss, Braunschweig, German) was used to examine the activity of *S. decolorationis* S12 after a 12-h reaction. A rapid fluorescence staining method using the LIVE/DEAD *Bac*Light™ Viability Kit (Molecular Probes Inc., Eugene, Oregon, USA) was applied to estimate both the viable and total counts of bacteria according to the manufacturer’s recommended protocol.

### TEM and SEM analyses

Transmission electron microscopy (TEM) and scanning electron microscopy (SEM) with energy dispersive X-ray spectroscopy (EDS) were used to observe the structures of the bacterial cells and the iron precipitates. Bacterial cell samples were collected for TEM analysis after a 12-h incubation, and Fe^0^ samples for SEM analysis were collected after a 30-h incubation. The suspended bacterial cells and Fe^0^ samples were collected separately. The cell pellet was separated from the medium by centrifugation at 12,000 × *g* for 5 min, and the pellet (containing cells and Fe^0^) was washed twice with 0.1 M phosphate buffer before fixation in 3% glutaraldehyde for 5 h. Then, the pellet was washed twice with phosphate buffer and dehydrated in a gradient series of ethanol solutions from 30 to 100% by incubation at each ethanol concentration for 15 min. For TEM analysis, the sample was treated with acetone and embedded in epoxy resin. Thin sections (80–90 nm) were cut with a diamond knife mounted on a Leica EM UC6 ultramicrotome and collected on carbon-coated Cu grids. TEM observation was performed with a JEOL JEM-2010HR TEM at 200 kV or a Hitachi H-7650 TEM at 80 kV. For SEM observation, the sample was treated with *tert*-butanol and then freeze dried. After sputtering with gold, the sample was deposited on the Cu carrier and observed with either a Hitachi H-3000 N SEM or a FEI Quanta 400 F SEM. The EDS measurement was performed with an Oxford INCA EDX spectrometer coupled with a JEOL JEM-2010HR TEM or an FEI Quanta 400 F SEM.

### Chemical analysis and calculation

The pH of the solution was measured using a digital multi-parameter 3430 meter (WTW, Germany). The Fe^2+^ concentration in the aqueous phase was measured using the HCl extraction ferrozine assay method as previously described [25]. The H_2_ concentration in the medium after a 12-h reaction was examined by hydrogen microelectrodes (model H_2_-50, Unisense A/S, Denmark) polarized at +1000 mV.

The decolorization of amaranth was measured by monitoring the decrease in absorbance at a wavelength of 520 nm with a Beckman DU640 UV/visible spectrophotometer. The decolorization rate and efficiency were calculated as follows:

$$ \mathrm{Decolorization}\ \mathrm{rate}\ \left(\%\right) = \left(A\hbox{--} B\right)/A \times 100\% $$

$$ \mathrm{Decolorization}\ \mathrm{efficiency}\ \left(\mathrm{mmol}\ {\mathrm{L}}^{\hbox{-} 1}\ {\mathrm{h}}^{\hbox{-} 1}\right) = \mathrm{Decolorization}\ \mathrm{rate} \times C/t $$

where *A* is the initial absorbance, *B* is the absorbance after the reaction, *C* is the initial concentration of amaranth (1 mM), and *t* is the decolorization reaction time (12 h). All assays were performed in triplicate.

Statistical analysis was conducted with Office Excel 2010, Origin V8.0, and SPSS V17.0 (SPSS Inc. Chicago, IL, USA) software. Treatment *P* values < 0.05 were considered significant.

## Results and discussion

### Effects of Fe^0^ on azo dye decolorization by strain S12

**Figure 1:**
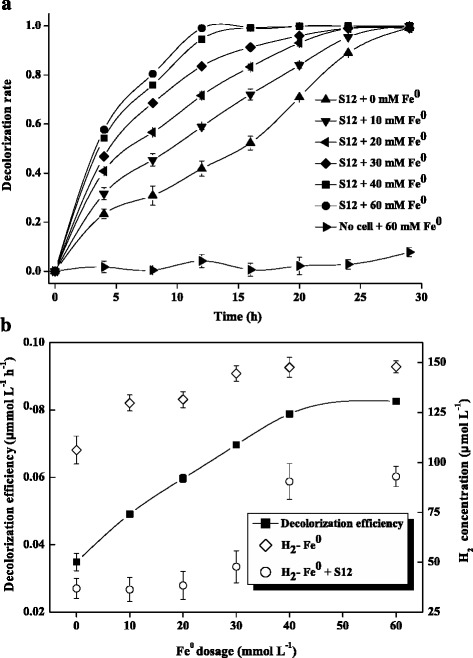
Effect of Fe^0^ dosage on the decolorization of azo dyes by *S. decolorationis* S12. (**a**) The time course of the decolorization rate at different Fe^0^ dosages. (**b**) Decolorization efficiency and H_2_ concentration at different Fe^0^ dosages after a 12-h reaction.

To confirm that Fe^0^ enhanced the anaerobic bioreduction of azo dye, decolorization by strain S12 was performed at six different dosages (0, 10, 20, 30, 40, and 60 mM) of Fe^0^ at pH 7.0 (Figure 1). The amaranth was completely decolorized in 30 h, except in the treatment without strain S12 cells, suggesting that strain S12 was the major azo reducer and that no observable direct reaction between Fe^0^ and amaranth occurred. In the presence of increasing doses of Fe^0^, the complete decolorization time for strain S12 rapidly reduced from 27 to 12 h, and the decolorization efficiency increased from 0.049 to 0.083 mmol L^−1^ h^−1^ (Figure 1). In addition, the decolorization efficiencies of the Fe^0^-added systems were much higher than those of the Fe^0^-free systems (0.035 mmol L^−1^ h^−1^), demonstrating that the addition of Fe^0^ enhanced the biodecolorization of azo dyes. This phenomenon might be attributable to the H_2_ supply from the cathodic corrosion of Fe^0^. Based on Equation (1), H_2_ is generated during the anaerobic corrosion of Fe^0^.

To determine if the H_2_ evolved from Fe^0^ can serve as an electron donor for strain S12 during the decolorization process, the concentration of H_2_ in the experimental systems after 12-h reactions with different Fe^0^ dosages was determined. As shown in Figure 1b, the H_2_ concentration increased with Fe^0^ dosage in both the presence and absence of strain S12 (from 36 to 93 μmol L^−1^ and from 129 to 148 μmol L^−1^, respectively), indicating that H_2_ was generated from the anaerobic corrosion of Fe^0^. However, the H_2_ concentrations in the presence of strain S12 were significantly lower than those measured in the cell-free tests (*P* < 0.001), suggesting that the produced H_2_ was consumed as an electron donor by strain S12 during azo reduction. In addition, as shown in Figure 1b, the dosage of Fe^0^ was a rate-limiting parameter for azo reduction when the dosage of Fe^0^ was less than 40 mM, which could be due to the limited H_2_ supply for strain S12. However, when the dosage of Fe^0^ was greater than 40 mM, the supply of H_2_ became adequate, and the decolorization efficiency reached a steady state. This study thus confirmed the presence of a dosage threshold for Fe^0^ (40 mM) for strain S12 in azo reduction. Previous study of the effect of Fe^0^ dosage on hexavalent chromium and carbon tetrachloride removal also reported a dosage threshold for Fe^0^ due to the H_2_ supply [26].

Furthermore, we also measured the Fe^2+^ concentrations in the absence and presence of strain S12 cells in the presence of 60 mM Fe^0^ (no rate limitation of azo reduction was observed at this dosage, as described above) because Fe^2+^ is one of the products of anaerobic Fe^0^ corrosion. After a 30-h incubation, the Fe^2+^ concentration in the experiments with strain S12 was 13.0 ± 0.5 μmol L^−1^, which was significantly higher than that of the abiotic control (2.1 ± 0.1 μmol L^−1^) (*P* = 0.03), suggesting that strain S12 promoted the dissolution of Fe^0^ particles. The same phenomenon was also observed by De Windt *et al*. [27] for anoxic Fe^0^ corrosion coupled with nitrate reduction by *S. oneidensis* MR-1. The mechanism of Fe^0^ consumption remains unclear. There were two likely explanations: (i) the consumption of H_2_ released from the Fe^0^ surface by strain S12 allowed the reaction described in Equation (1) to occur and shift to the right, or (ii) strain S12 reacted with Fe^0^ directly (such as from the uptake of iron discussed below) and accelerated the transformation of iron. The data in this study indicate that both explanations are correct and complementary, but more work is needed to clarify the exact mechanism.

**Figure 2:**
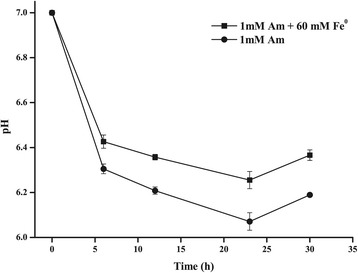
Changes in pH during the biodecolorization process with/without Fe^0^.

To further examine the changes in the microenvironment around strain S12 and Fe^0^, we detected pH changes during the biodecolorization process in the absence and presence of 60 mM Fe^0^ conditions with an initial strain S12 cells of 10^7^ CFU/ml (Figure 2). Generally, the pH value decreased in the decolorization process both in the absence and presence of Fe^0^ conditions, with the lowest pH values of 6.07 and 6.25 at 24 h, respectively. This decrease in pH is likely due to the production of acetate by lactate oxidation as well as proton release during azo reduction [28,29]. Notably, higher pH values were observed in the systems with Fe^0^ than in the Fe^0^-free tests (*P* = 0.047), which could be due to a buffering effect of OH^−^ from the corrosion of Fe^0^ based on Equation (1). Slight changes in the microenvironmental pH around the cells can lead to variations in the activity and stability of enzymes and proteins (such as azoreductase) participating in growth and decolorization [30,31], implying that the improved pH buffer capability of the Fe^0^-microbial system may also contribute to enhance azo reduction. Similar results have been reported in other Fe^0^-assisted biodecolorization studies [11].

### Shifts in the size distribution pattern of Fe^0^ particles during biodecolorization

**Figure 3:**
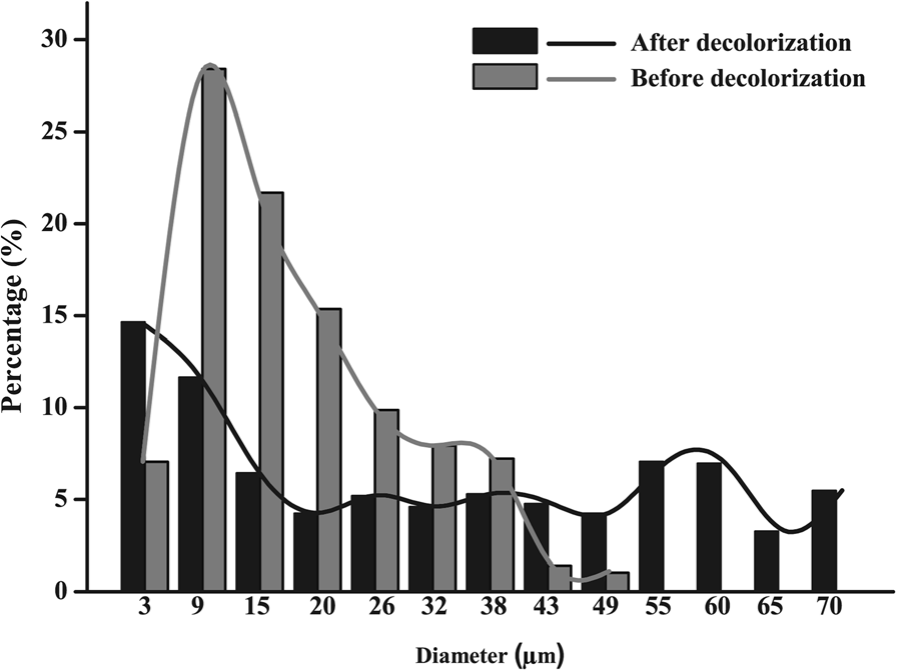
Shifts in the size distribution pattern of the Fe^0^ particles during the biodecolorization process.

To measure the consumption of Fe^0^ during biodecolorization, the change in the size distribution of the Fe^0^ particles before and after decolorization was characterized using dynamic light scattering (DLS) in the systems inoculated with strain S12 and 60 mM Fe^0^ at pH 7.0 (Figure 3). The percentages of small-sized (<9 μm) and large-sized (43–70 μm) iron particles dramatically increased from 7.06% to 17.47% and 2.44% to 37.89%, respectively, while the percentage of medium-sized particles (9–43 μm) decreased from 90.50% to 44.62% after decolorization. This trend was consistent with the SEM analysis of the particles, which revealed that after a 30-h biological azo reduction, the medium-sized particles with rough surfaces disappeared, and the large-sized particles with smooth surfaces formed clusters (Additional file 1: Figure S1a and Additional file 1: Figure S1b). The surfaces of the Fe^0^ clusters were further analyzed by EDS, and Fe, O, P, and C were detected as the primary elements (Additional file 1: Figure S1c). According to Equation (1), this result may be due to the formation of ferrous iron precipitates (e.g., Fe(OH)_2,_ Fe_3_(PO_4_)_2_, and FeCO_3_) that attached to the surface of Fe^0^ [32,33]. These observations confirmed that partially solid Fe^0^ was consumed during the biodecolorization process.

### Effects of Fe^0^ on cell morphology and viability

**Figure 4:**
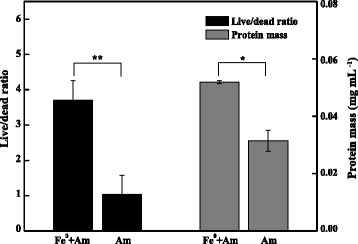
Live/dead ratios and protein masses of bacteria with/without Fe^0^. For the T-test, *, *P* value < 0.05; **, *P* value < 0.01.

**Figure 5:**
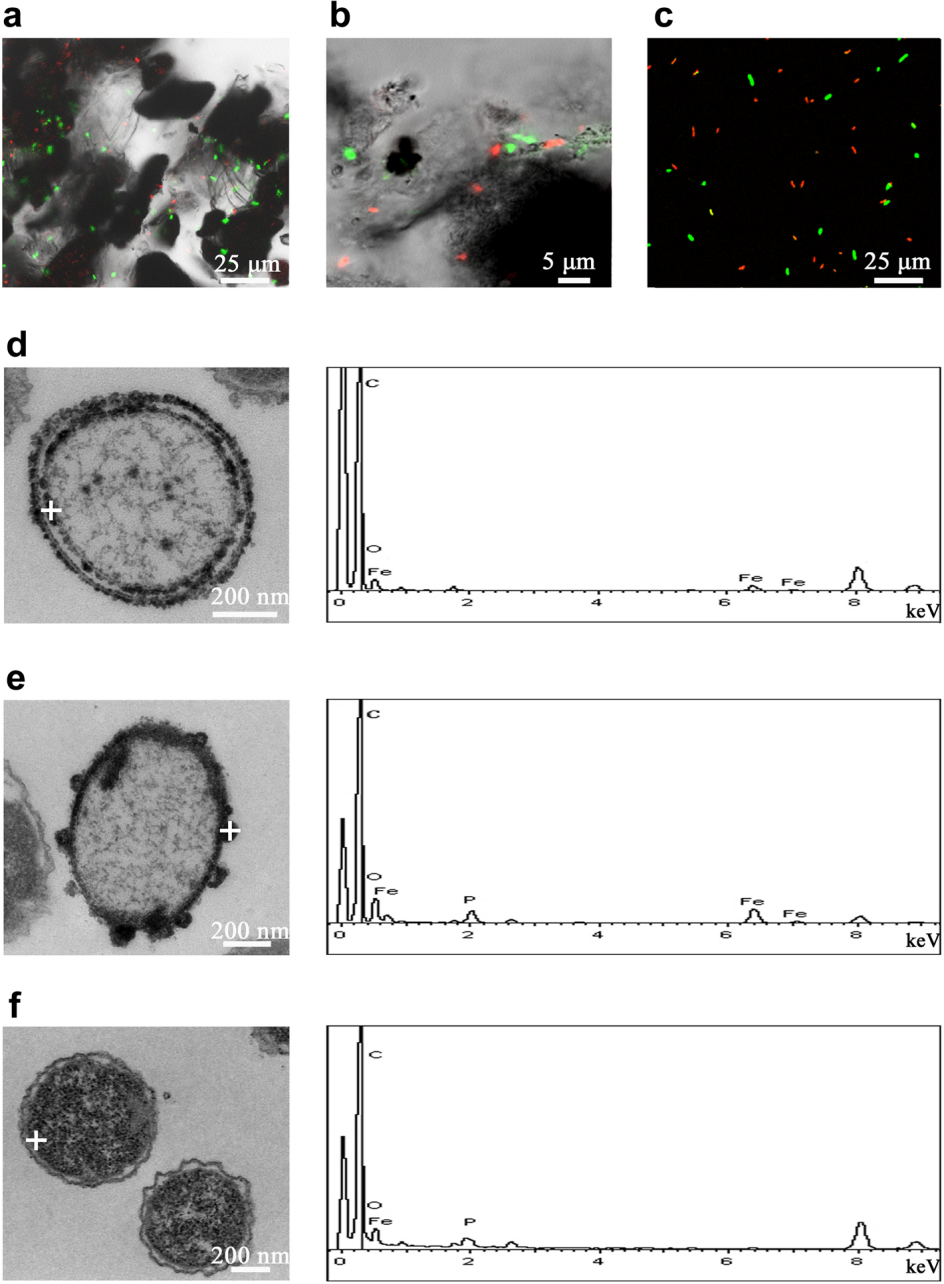
Laser scanning confocal microscope (LSCM) and transmission electron microscopy/energy dispersive X-ray spectroscopy (TEM/EDS) analysis of strain S12 cells. (**a**) and (**b**): LSCM images of cells after biodecolorization for 12 h with 60 mM Fe^0^. The red point represents dead cells, and the green point represents live cells. The iron particles are indicated in black. (**c**): LSCM image of cells after biodecolorization without Fe^0^. (**d**) and (**e**): TEM images and EDS profiles of cells after biodecolorization for 12 h with 60 mM Fe^0^, showing the presence of iron precipitates inside and outside the cell membrane. (**f**): TEM images and EDS profiles of cells after biodecolorization without Fe^0^. The white cross is the site of EDS analysis. The white short line is a scale bar, and the corresponding length is on the top of the scale bar.

We observed that the growth and viability of strain S12 were affected by added Fe^0^, and we investigated the mechanism by which Fe^0^ significantly promoted azo reduction. To evaluate cell growth, we measured the biomass yield (i.e., the protein mass) in the presence of 60 mM Fe^0^ and in the Fe^0^-free control after cultivation for 12 h. The protein mass in the Fe^0^-supplemented culture was nearly twice more than that in the culture without Fe^0^, indicating that added Fe^0^ can promote the growth of strain S12 (*P* < 0.05) (Figure 4). As an additional electron donor provided by Fe^0^ corrosion, H_2_ likely contributed to this growth promotion. The bacterial cell morphology and viability after incubation with and without Fe^0^ (60 mM) for 12 h were evaluated by CLSM and TEM. CLSM analysis revealed more live (green) cells in the presence of Fe^0^ than in the control (Figure 5a versus Figure 5c). The live/dead ratio of strain S12 in the Fe^0^ culture was approximately 3.5-fold higher than that in the culture without Fe^0^ (*P* < 0.01), indicating that the presence of Fe^0^ was beneficial for maintaining cell viability (Figure 4). CLSM also revealed that strain S12 cells were attached to the surfaces of the micro-scale Fe^0^ particles (Figure 5a,b), suggesting that strain S12 decolorized azo dyes in both the aqueous and solid phases. Bacterial cell appendages (e.g., the flagella and pili), which were observed in strain S12 (Additional file 1: Figure S2) and confirmed by the genome sequence [34], played an important role in adhesion to the micro-scale Fe^0^ particles. Other *Shewanella* spp., such as *S. alga*, *S. putrefaciens* and *S. oneidensis*, also grow adhering to iron minerals [35-37].

The TEM/EDS images demonstrated differences in cell morphology in the Fe^0^-supplemented culture (Figure 5d,e) and the control (Figure 5f). In the Fe^0^-supplemented culture, extracellular and/or intracellular fine-grained Fe precipitates or clusters formed outside and/or inside the cell membrane (with mean sizes of 80.52 ± 15.82 nm and 25.90 ± 6.53 nm, respectively) (Figure 5d,e). These results indicated direct interactions between Fe^0^ and strain S12, providing good evidences for microbial-driven biogeochemical cycling. These direct interactions promoted the transformation of iron as well as the removal of contaminants. First, for contaminant removal, Fe precipitates provided more Fe sources for the synthesis of enzymes and thus enhanced microbial activity. Elemental iron assimilated by strain S12 was an active ingredient for multiple dehydrogenases and hydrogenases (such as [Fe-S] cluster-containing lactate dehydrogenase and [Ni–Fe] and [Fe–Fe] catalytic site-containing hydrogenases), ensuring higher cell physiological and azoreductive activities [14,34,38]. Second, strain S12 cells, which were attached to the surface of Fe^0^, coupled the consumption of H_2_ (released from Fe^0^ corrosion) and the reduction of azo dye by azo respiration. For iron transformation, the process included several steps as follows: (i) Fe^2+^ released from Fe^0^ corrosion and reversibly adhered to the surface of Fe^0^ or strain S12 cells; (ii) Fe^2+^-containing precipitates formed (e.g., Fe(OH)_2,_ Fe_3_(PO_4_)_2_, FeCO_3_) when anions presented, and (iii) the small size and high surface-to-volume ratio of fine-grained particles (Fe^0^-Fe^2+^_[solid]_) enabled significant adsorption of Fe precipitates to the outer membrane of strain S12 cells and the intracellular uptake of Fe precipitates to the cytoplasm of strain S12 cells. Fe^0^ corrosion was accelerated because the concentration of Fe^2+^_[aqueous]_ was diluted by strain S12 through steps (i) and (iii), suggesting that strain S12 contributed to the transformation of iron, although a quantitative analysis could not be performed here. The biosorption of Fe precipitates has also been observed for other *Shewanella* spp. [27] and *Dehalococcoides* spp. [39] involved in Fe^0^-assisted bioremediation, revealing universal direct interactions between bacteria and minerals during containment removal [40].

### Mechanisms of Fe^0^-assisted biodecolorization

**Figure 6:**
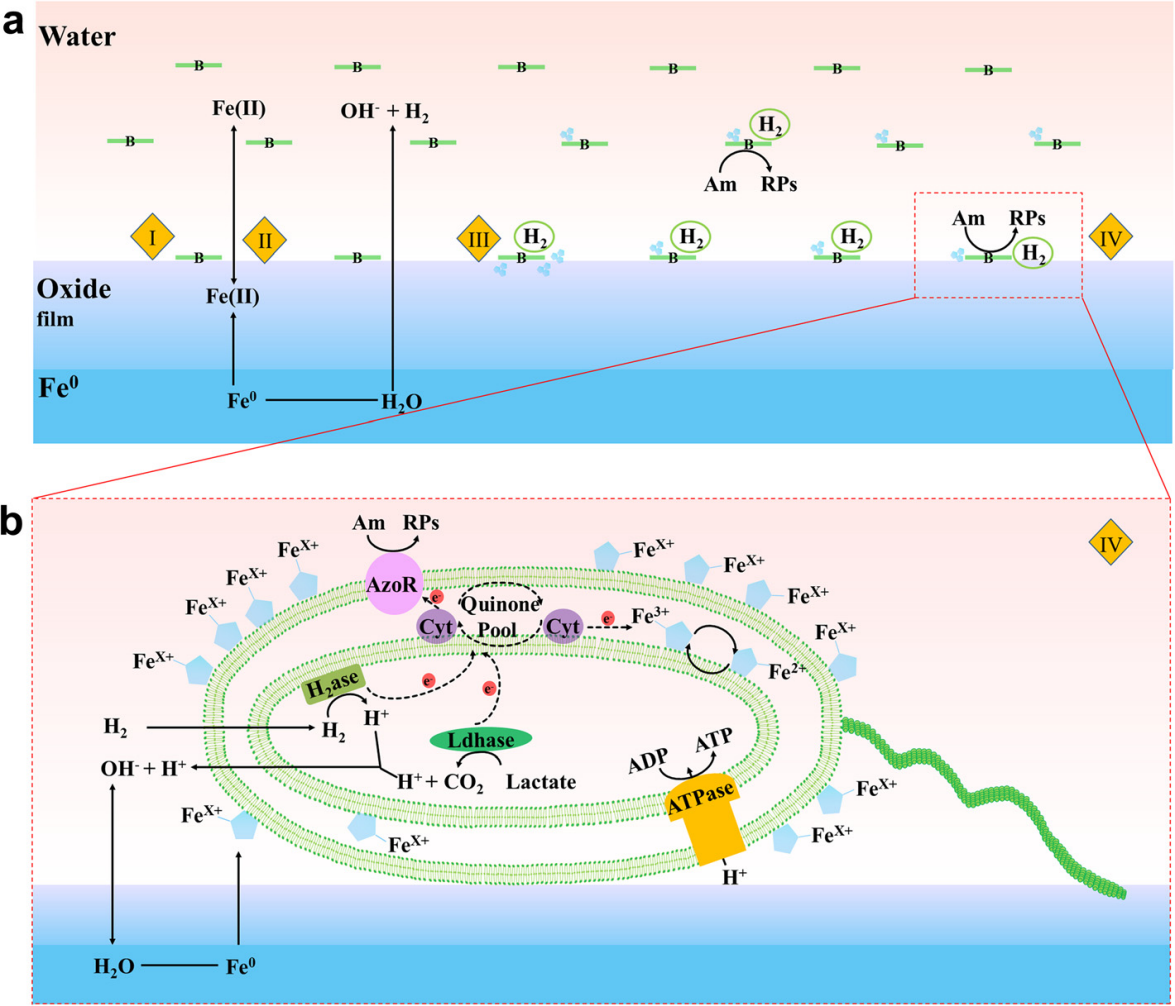
Proposed model for Fe^0^-assisted azo decolorization by *S. decolorationis* S12. (**a**) Four sections (I, II, III, and IV) of Fe^0^-assisted azo biodecolorization. (**b**) Magnification of section IV in (**a**). Note: B = bacterium; Am = amaranth; RPs = reductive products of amaranth; AzoR = azoreductase; H_2_ase = hydrogenase; Ldhase = lactate dehydrogenase; Cyt = cytochrome protein.

We propose a conceptual model for the Fe^0^-assisted azo dye reduction process by *S. decolorationis* S12 (Figure 6). In the model, four sections (I, II, III, and IV) explain the mechanism. In section I, the cells of strain S12, which use the azo bond as the terminal acceptor to complete anaerobic respiration, attach to the surface of the micrometer-scale iron particles. In section II, Fe^0^ reacts with H_2_O in a neutral-pH, anaerobic environment and produces H_2_ and OH^−^. Strain S12 cells utilize H_2_ as an electron donor and energy source for growth. In addition, the OH^−^ generated from Fe^0^ erosion neutralizes the H^+^ released from bacterial metabolism, maintaining the proper pH for microbial growth. In section III, the strain S12 cells accelerate the dissolution of iron particles due to the consumption of H_2_, resulting in the generation of nanometer-scale particles and a higher concentration of Fe^2+^. In section IV, nanometer-scale Fe precipitates adsorb to the surface of the outer membranes and are transported inside the strain S12 cells, improving cell growth and maintenance of viability. The cellular physiological analyses in this study suggest that the addition of Fe^0^ enhances protein content and bacterial activity. Potential functional proteins and pathways for bacterial azo respiration in the presence of Fe^0^ are shown in Figure 6b, although further work is needed to verify their involvement.

## Conclusions

The results of batch experiments of azo decolorization by combining the use of Fe^0^ and the azo-reducing bacterium *S. decolorationis* strain S12 expanded our knowledge of the enhancement of microbial azo reduction by Fe^0^. A conceptual model for Fe^0^-assisted microbial azo reduction was established based on the direct and indirect interactions between microbes and Fe^0^ after characterizing the changes in the morphology of the Fe^0^ particles, the physiological activities of the bacteria, and the physicochemical properties of the azo reduction system. This model will facilitate the development of azo dye remediation technology. However, to further elucidate the mechanisms underlying these processes, transcriptomic and proteomic analyses should be performed to track the dynamics and adaptive responses of strain S12 during Fe^0^-assisted azo reduction processes.

## Additional file

Additional file 1: Figure S1. Scanning electron microscope/energy dispersive X-ray spectroscopy (SEM/EDS) analysis of the iron particles. (a) Iron particles before biodecolorization. (b) Iron particles after biodecolorization for 30 h. The yellow cross is the site (containing the slice structure) of EDS analysis. (c) The EDS profile of the slice structure on the iron particle surface after biodecolorization. In (a) and (b), the white short line is a scale bar, and the corresponding length is on the top of the scale bar. **Figure S2.** Transmission electron microscopy (TEM) image of *S. decolorationis* S12 with a flagellum. The white short line is a scale bar, and the corresponding length is on the top of the scale bar. The white arrow indicates a flagellum on the polar surface of the *S. decolorationis* S12 cell.
