# Supplementary material for: Characterization of the enhancement of zero valent iron on microbial azo reduction
Source: BMC Microbiol. 2015 Apr 10;15:85. doi: 10.1186/s12866-015-0419-3 (PMC4428006; doi:10.1186/s12866-015-0419-3)
Supplement: Additional file 1: Figure S1. — Scanning electron microscope/energy dispersive X-ray spectroscopy (SEM/EDS) analysis of the iron particles. (a) Iron particles before biodecolorization. (b) Iron particles after biodecolorization for 30 h. The yellow cross is the site (containing the slice structure) of EDS analysis. (c) The EDS profile of the slice structure on the iron particle surface after biodecolorization. In (a) and (b), the white short line is a scale bar, and the corresponding length is on the top of the scale bar. Figure S2. Transmission electron microscopy (TEM) image of S. decolorationis S12 with a flagellum. The white short line is a scale bar, and the corresponding length is on the top of the scale bar. The white arrow indicates a flagellum on the polar surface of the S. decolorationis S12 cell. [file 12866_2015_419_MOESM1_ESM.docx]

**Additional files**

**
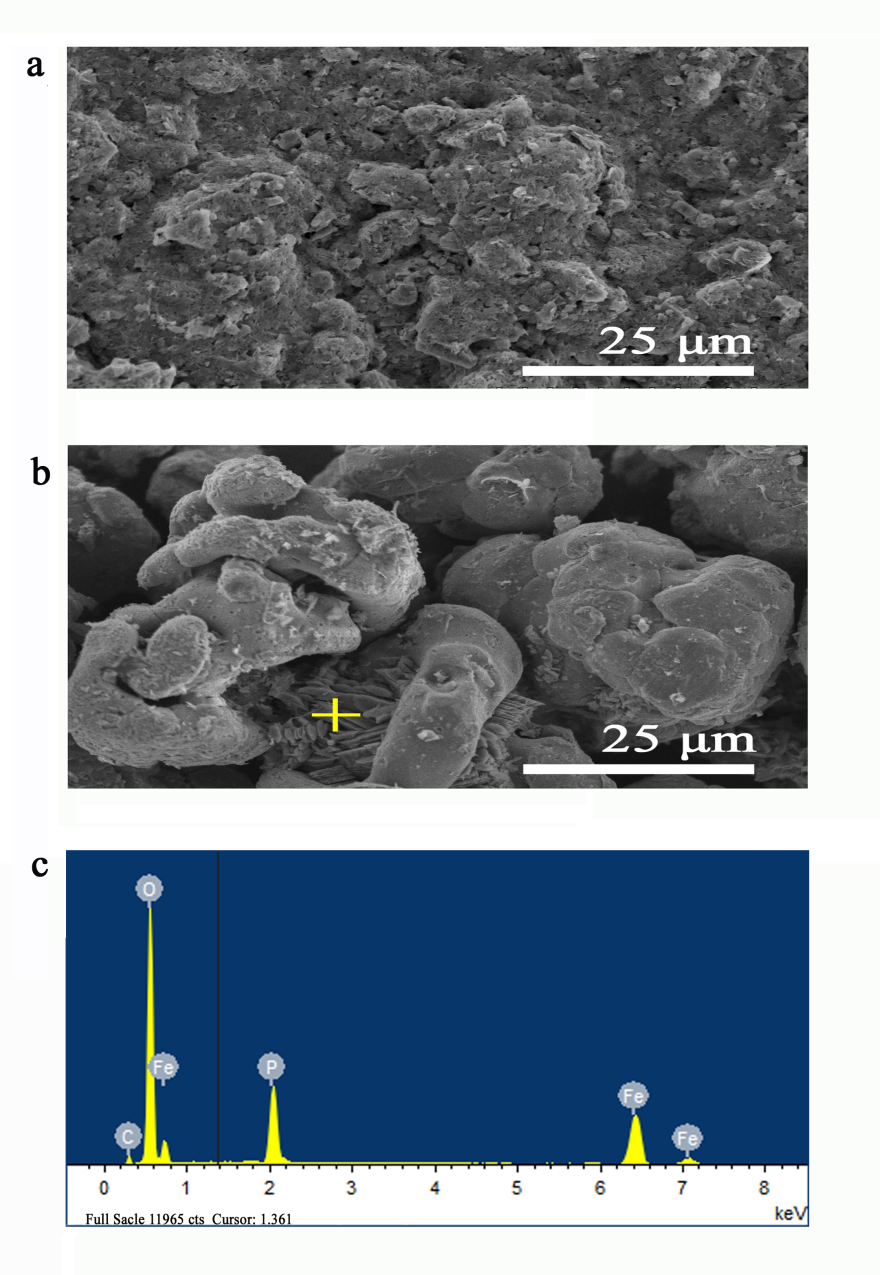
**

**Fig. S1** Scanning electron microscope/energy dispersive X-ray spectroscopy (SEM/EDS) analysis of the iron particles. (a) Iron particles before biodecoloriztion. (b) Iron particles after biodecolorization for 30 h. The yellow cross is the site (containing slice structure) for EDS analysis. (c) The EDS profile of the slice structure on the iron particle surface after biodecolorization. In (a) and (b), white short line is a scale bar, and the corresponding length is on the top of the scale bar.


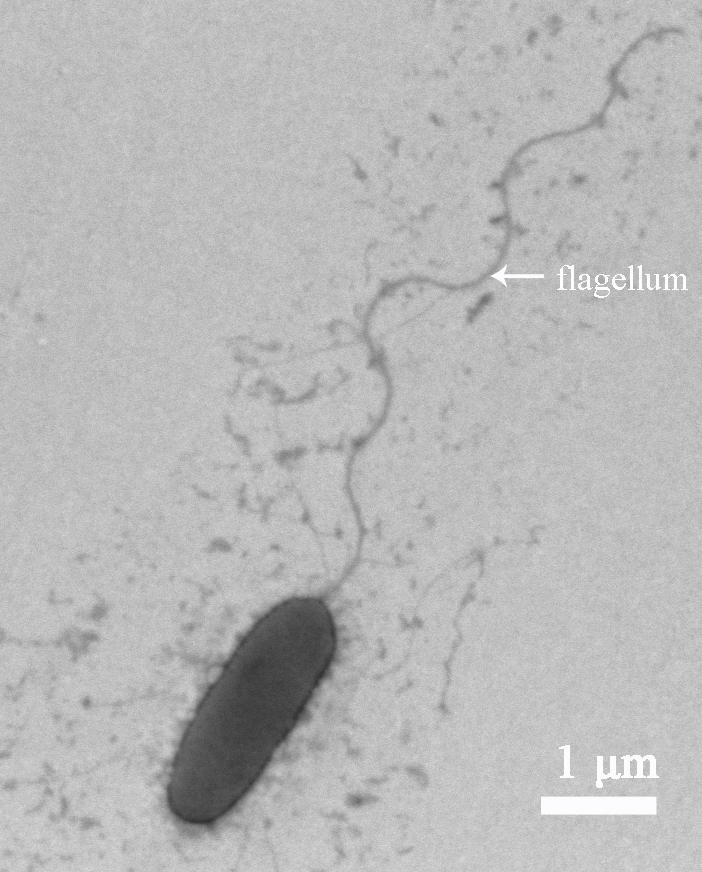


**Fig. S2** Transmission electron microscopy (TEM) image of *S. decolorationis* S12 with a flagellum. White short line is a scale bar, and the corresponding length is on the top of the scale bar. The white arrow indicates a flagellum on the polar of the *S. decolorationis* S12 cell.
